# A Novel Mouse Dscam Mutation Inhibits Localization and Shedding of DSCAM

**DOI:** 10.1371/journal.pone.0052652

**Published:** 2012-12-26

**Authors:** R. Dee Schramm, Shuai Li, Belinda S. Harris, Ryan P. Rounds, Robert W. Burgess, F. Marty Ytreberg, Peter G. Fuerst

**Affiliations:** 1 Department of Biological Sciences, University of Idaho, Moscow, Idaho, United States of America; 2 The Jackson Laboratory, Bar Harbor, Maine, United States of America; 3 Department of Physics, University of Idaho, Moscow, Idaho, United States of America; 4 University of Washington WWAMI Medical Education Program, University of Idaho, Moscow, Idaho, United States of America; Academia Sinica, Taiwan

## Abstract

The differential adhesion hypothesis of development states that patterning of organisms, organs and tissues is mediated in large part by expression of cell adhesion molecules. The cues provided by cell adhesion molecules are also hypothesized to facilitate specific connectivity within the nervous system. In this study we characterize a novel mouse mutation in the gene *Dscam* (Down Syndrome Cell Adhesion Molecule). Vertebrate DSCAM is required for normal development of the central nervous system and has been best characterized in the visual system. In the visual system DSCAM is required for regulation of cell number, mosaic formation, laminar specificity, and refinement of retinal-tectal projections. We have identified a novel mutation in *Dscam* that results in a single amino acid substitution, R1018P, in the extracellular domain of the DSCAM protein. Mice homozygous for the R1018P mutation develop a subset of defects observed in *Dscam* null mice. In vitro analysis identified defects in DSCAM^R1018P^ localization to filopodia. We also find that wild type DSCAM protein is constitutively cleaved and shed from transfected cells. This secretion is inhibited by the R1018P mutation. We also characterized a novel splice isoform of *Dscam* and identified defects in lamination of type 2 and type 6 cone bipolar cells in *Dscam* mutant mice. The identification and characterization of partial loss of function mutations in genes such as *Dscam* will be helpful in predicting signs and symptoms that may be observed in human patients with partial loss of DSCAM function.

## Introduction

Identifying the mechanisms by which cells differentiate into complex tissues is a central goal of developmental biology. The nervous system is a particularly difficult and exciting system in which to study development because of its complexity. The nervous system is composed of a very large number of cell types that make specific connections to a limited number of other cell types. Strong evidence suggests that the generation of distinct neural cell types is mediated through differential expression of transcription factors. These transcription factors execute expression of genetic programs that specify cell type. The specificity of connections that neurons make is then mediated by the production and recognition of extracellular cues. For example, netrins guide axons to their targets through interactions with various receptors [1].

Much initial work directed at understanding how connectivity within the nervous system develops focused on a simple system, the neuromuscular junction, and proteins such as Agrin and Musk that are essential for innervation of skeletal muscle [2], [3], [4]. The retina is another popular model system for understanding neural connectivity. The retina offers a more complicated system in which to study connectivity, containing a variety of neuron-neuron synapses, electrical synapses and organized circuits. The limited number of cell types and availability of transgenic models and antibody reagents, has also made the retina a focus of developmental neurobiologists. The retina is organized in both a top to bottom vertical field and a horizontal dorsal-ventral-lateral-medial field. Vertically the retina is organized into circuits that collect, process and transmit visual information to the rest of the brain. These circuits are specialized to detect different aspects of vision, such as color, movement and edges. Many types of retinal neurons are spaced in a non-random fashion across the horizontal plane of the retina. This spacing, referred to as mosaic spacing, is thought to ensure that a given portion of the visual world is sampled by most or all of the aforementioned specialized circuits [5].

Identification of genes that specify spacing and connectivity of retinal neurons has begun to produce a more complete picture of how the retina is organized. A combination of molecules acts to guide neurons towards making appropriate contact through differential adhesion. Differential adhesion involves the production of both adhesive cues and repulsive or indifference cues. Neuroligins and neurexins are among the best-characterized adhesive cues and their differential expression at the proto-synapses of pre and postsynaptic cells help to facilitate the specificity of neural connectivity [6]. Differential adhesion also involves cues that specify avoidance, indifference or repulsion. The semaphorins and plexins, for example, guide the targeting of neurites within the retinal innerplexiform layer to specific depths. Semaphorins and plexins also prevent fasciculation of the neurites of some cell types in which they are expressed [7], [8], [9]. Proteins such as MEGF10 and MEGF11 also mediate avoidance and are required in order to facilitate horizontal spacing of cholinergic and horizontal cells within the retina, while the gamma-protocadherin complex mediates isoneuronal avoidance between the processes of a single cell [10], [11].

The immunoglobulin superfamily adhesion molecules *Dscam* and *Dscaml1* also function in preventing adhesion [12], [13]. *Dscam* and *Dscaml1* are required for multiple aspects of retinal development, including mosaic patterning of cell types, prevention of fasciculation, regulation of cell number and laminar organization. DSCAM is produced in a large number of spatially overlapping cell types that all independently require DSCAM to prevent adhesion, but maintain their homotypic identity in its absence, in that they adhere to like cells. This suggests that the simple and elegant model proposed for MEGF protein function, in which a homophilic adhesion molecule mediates avoidance of cells within a cell type from like cells, is insufficient to explain DSCAM function. Current models therefore invoke DSCAMs acting as part of a larger adhesion code. In this model some as yet unidentified molecules act as cell type identifiers, while DSCAMs are required to prevent these cell types from adhering homotypically [14].

In this study we characterize function of the DSCAM protein in retinal development using a novel *Dscam* point mutation. We find that the mutation, which results in a single amino acid substitution, causes a subset of the *Dscam* null phenotypes. Through in vitro characterization we find that DSCAM is targeted to growth cone filopodia and post-translationally cleaved. We also find that the ectodomain of the protein is shed. The point mutation allele blocks targeting to filopodia and secretion. In vivo, the mutant protein accumulates in the cell body or retinal neurons suggesting that the mutation prevents proper targeting of the protein. Our findings suggest a model in which wild type DSCAM can act as a diffusible ligand.

## Results

### A *Dscam* Point Mutation

**Figure 1:**
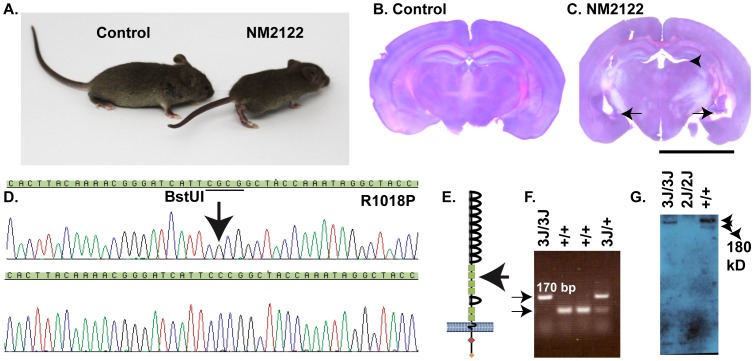
Amino Acid substitution R1018P is genetic basis of nm2122. A , A spontaneous mutation, nm2122, occurred at The Jackson Laboratory and exhibited overt phenotypes, including a large dome shaped head and muscle stiffness, similar to previously characterized *Dscam* mutants. **B** and **C**, nm2122 mutant mice develop enlarged central and lateral ventricles compared to controls (arrows). **D**, After complementation tests with another *Dscam* mutant, NM992 (*Dscam^2J^*), failed, the *Dscam* open reading frame was sequenced and a single nucleotide substitution was found resulting in substitution of proline at 1018 in place of the wild type arginine. The nm2122 mutation will henceforth be referred to as *Dscam^3J^*. **E**, The *Dscam^3J^* mutation is located in the second fibronectin domain (arrow). **F**, The mutation destroys a recognition site for the enzyme BstUI, allowing wild type, heterozygous and homozygous mutants to be identified based on PCR of the mutation-containing region followed by subsequent restriction enzyme digest. The wild type allele is digested by BstUI resulting in two bands of close to equivalent size, while the mutant allele remains intact. **G**, Western blot analysis of wild type, *Dscam^2J^* and *Dscam^3J^*. A polyclonal antibody to the N-terminus of DSCAM recognizes a single band of approximately 220 kD and a slightly smaller band in retinal extracts prepared from wild type mice while a single band of 220 kD was detected in *Dscam^3J^* extracts. No protein product is observed from retina (not shown) or brain extract prepared from *Dscam^2J^* mice. The scale bar in (C) is equivalent to 1 cm.

A spontaneous recessive neurological mutation, nm2122, arose at The Jackson Laboratory on an inbred stock of C3H/Smn.C-Prkdc<scid> mice. Nm2122 presented an array of mutant phenotypes, including kyphosis, hydrocephalus and apparent vestibular defects (Figure 1 A–C). Based on the presentation of these phenotypes, and their initial mapping to the distal end of Chromosome 16, a complementation test was established between nm2122 and *Dscam^2J^* (nm992), a mutant mouse line that carries a loss of function allele of *Dscam* [15], [16]. Out of 26 pups, five had phenotypes similar to that of nm2122 and *Dscam^2J^*, indicating that *Dscam^2J^* failed to complement nm2122 and that the mutations are allelic.

The nm2122 *Dscam* open reading frame was sequenced from whole brain cDNA and a single nucleotide substitution, guanine to cytosine at nucleotide 3052 of the *Dscam* transcript, was identified (Figure 1D). This mutation results in substitution of a proline residue at amino acid 1018 in place of the wild type arginine in the second fibronectin domain of DSCAM (Figure 1E). The nm2122 mutation will be henceforth referred to as *Dscam^3J^*, because it is the third spontaneous *Dscam* mutation identified at The Jackson Laboratory, while the mutant protein will be referred to as DSCAM^R1018P^. A PCR RFLP genotyping technique was developed to genotype *Dscam^3J^* mice. Primers amplify the region containing the *Dscam^3J^* mutation from genomic DNA, which is then digested with the enzyme BstUI. The *Dscam^3J^* mutation destroys a BstUI site, and DNA amplified from mutant alleles retains a large undigested band after digestion, whereas DNA products amplified from wild type alleles result in a digested product (Figure 1F). Western blot analysis (WBA) was performed to determine if DSCAM protein was made in *Dscam^3J^* mutant mice. WBA of protein extract from wild type postnatal day 15 (P15) cerebellum resulted in a bright band of approximately 220 kD in size and a faint band of slightly smaller size. A single band of approximately 220 kD in size was detected in *Dscam^3J^* extracts, while no band was observed in protein extracts from *Dscam^2J^* mice (Figure 1G). Similar WBA results were obtained from these genotypes using protein extracts generated from retina, cortex and olfactory bulb (data not shown).

### Characterization of Mutant Phenotypes in *Dscam^3J^* Mice

**Figure 2:**
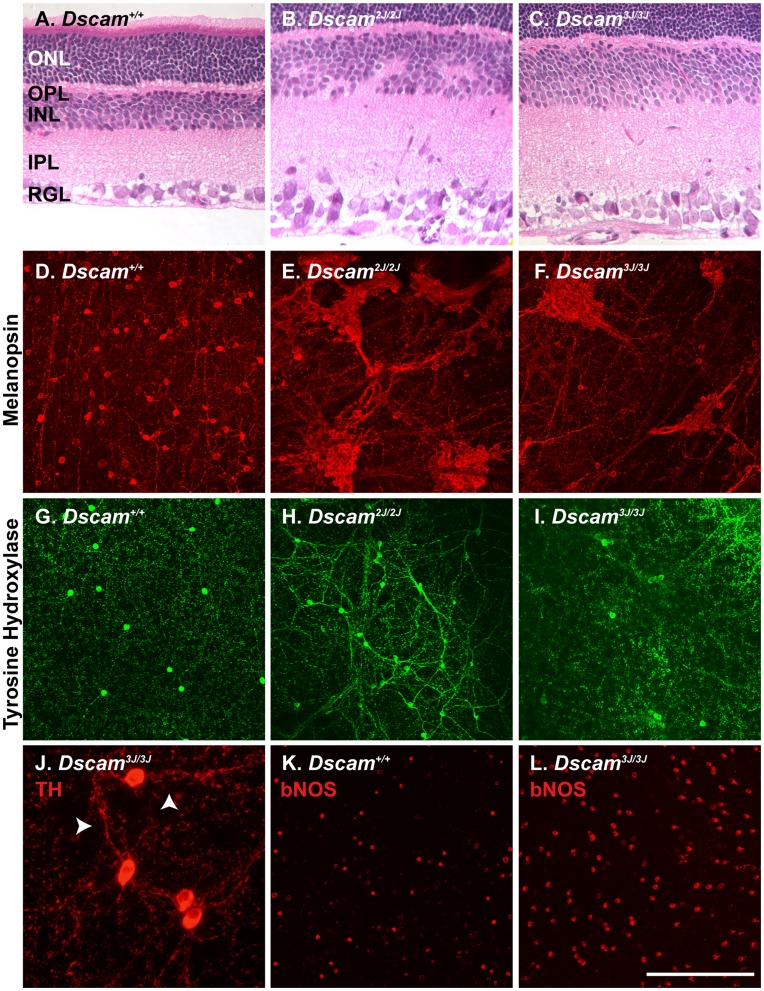
*Dscam^3J^* mutation reproduces some aspect of *Dscam* null retina. A –**C**, Retinal sections from wild type, *Dscam^2J^* and *Dscam^3J^* mice were stained with hematoxylin and eosin. In the wild type retina (A) the three cellular layers are neatly stacked and the synapse containing plexiform layers do not intrude within the cellular layers. **B**, Cell number is increased in the *Dscam^2J^* mutant retina, with ectopic cells located in the inner plexiform layer, which projects into the inner nuclear layer. **C**, The *Dscam^3J^* retina is hypercellular; however, cellular lamination is more neatly organized compared to the *Dscam^2J^* retina. **D**–**F**, Retinal ganglion cell spacing and arborization is disrupted in both the *Dscam^2J^* and *Dscam^3J^* retina compared to wild type. **G**–**I**, Amacrine cell spacing and arborization is disrupted in the *Dscam^2J^* mutant retina but this degree of disruption is not observed in the *Dscam^3J^* mutant retina (**I**). **J**, Occasional loose fasciculation of *Dscam^3J^* dopaminergic cell neurites was observed (arrows). **K** and **L**, Wild type and *Dscam^3J^* retinas were stained with antibodies to bNOS, to detect bNOS-positive amacrine cells. The number of bNOS positive amacrine cells is increased in the *Dscam^3J^* retina. The scale bar in (A-C) is equivalent to 132 µm. The scale bar in (I) is equivalent to 320 µm in D-I and K and L, and 100 µm in J.

The inbred line that the *Dscam^3J^* allele arose on carries the recessive retinal degeneration allele of *Pde6b*, rd1. *Pde6b^rd1^* results in rapid degeneration of photoreceptors around the time of eye opening. In order to examine retinal structure of *Dscam^3J^* mice, the line was crossed to an inbred C3H line that carries a wild type allele of *Pde6b* [17]. Other lines of *Dscam* mutant mice have disrupted laminar specificity, arborization and spacing of retinal neurons. We assayed these phenotypes in *Dscam^3J^* mice. *Dscam^2J^* mice were used as negative controls because they are on a similar genetic background and because *Dscam^del17^* mice, the first published *Dscam* mouse mutant, make a small amount of residual protein as a result of an alternative splice form (Figure S1) [12]. We first assayed gross retinal lamination by staining sections of wild type, *Dscam^2J^* and *Dscam^3J^* retina with hematoxylin and eosin (H&E). The *Dscam^3J^* allele resembled the protein null *Dscam^2J^* allele in that the size of the inner plexiform, inner nuclear and retinal ganglion cell layers were expanded. Unlike the *Dscam^2J^* inner nuclear layer, which contains fascicles of processes, the *Dscam^3J^* inner nuclear layer is evenly laminated (Figure 2 A–C). This suggested that the amacrine cells of the *Dscam^3J^* inner nuclear layer may be less disrupted than the amacrine cells of the *Dscam^2J^* retina. We therefore stained amacrine and ganglion cell populations in whole retinas to examine their neurite arborization and soma spacing. Arborization and soma spacing defects were observed in intrinsically photosensitive retinal ganglion cells (ipRGCs), a population that is highly sensitive to *Dscam* dosage [18], [19] (Figure 2 D–F). Less extensive defects were observed in the arborization and spacing of dopaminergic amacrine (DA) cells in *Dscam^3J^* retinas compared to *Dscam^2J^* retinas (Figure 2 G–I). An increase in the incidence of juxtaposed DA cells was observed in the *Dscam^3J^* retina compared to wild type or random simulations matched for cell number (Average 5.15 pairs per retina in simulations versus 19 in *Dscam^3J^* retinas T-Test = <0.01). Loose fasciculation of DA cell neurites was occasionally observed in the *Dscam^3J^* retina (Figure 2J; arrows). Similar defects in cell number and spacing were observed in bNOS amacrine cells (Figure 2K and L). bNOS positive amacrine cells also appeared hypertrophied in the *Dscam^3J^* mutant retina, in a similar fashion to the hypertrophy observed in some DscamL1-deficient cell types [13]. Therefore, defects in cell number are conserved between *Dscam^3J^* and other *Dscam* mutant alleles, but a lesser disruption in arborization and spacing of amacrine cells was observed.

**Figure 3:**
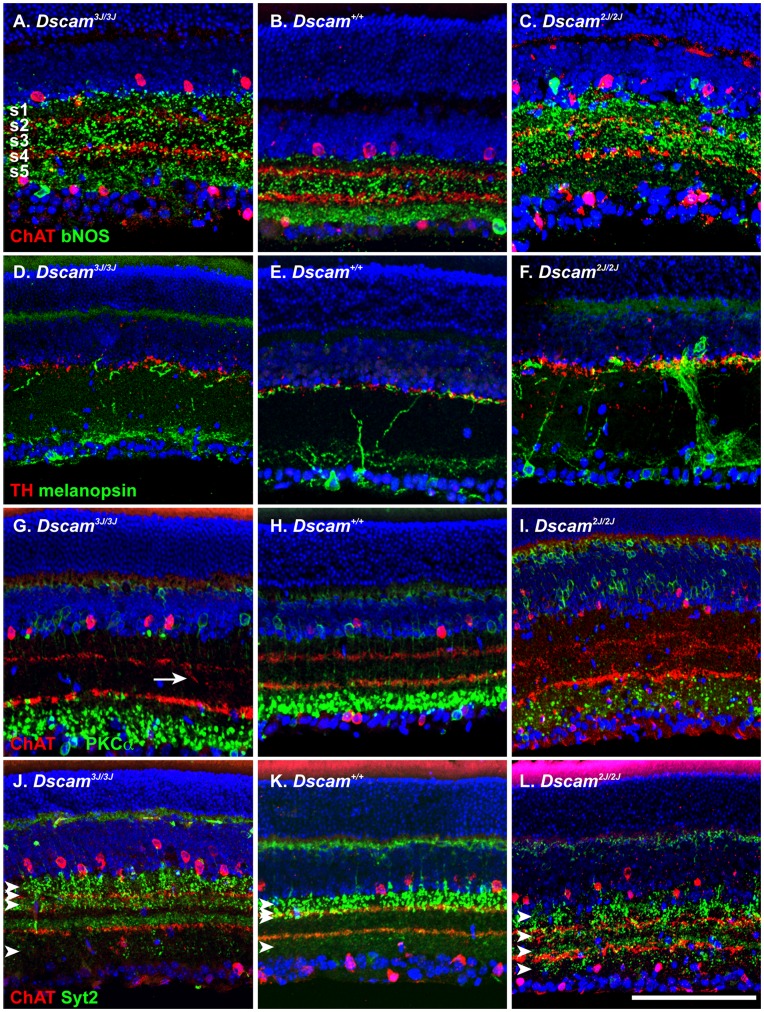
Neurite laminar specificity is preserved in *Dscam^3J^* mutant retina. A –**C**, Wild type, *Dscam^2J^* and *Dscam^3J^* retinal sections were stained with antibodies to ChAT, a marker of starburst amacrine cells and bNOS, a marker of bNOS-amacrine cells (A–C). Wild type laminar specificity of bNOS-positive neurites in s1, s3 and s5 is roughly preserved in the *Dscam^3J^*, but not *Dscam^2J^* retina. **D**–**F**, Wild type, *Dscam^2J^* and *Dscam^3J^* retinal sections were stained with antibodies to TH, a marker of dopaminergic amacrine cells and melanopsin, a marker of melanopsin-positive retinal ganglion cells. Although extensive fasciculation of neurites is observed, the laminar specificity pattern of cell types is maintained in mutant genotypes. **G**–**H**, Wild type, *Dscam^2J^* and *Dscam^3J^* retinal sections were stained with antibodies to ChAT, a marker of starburst amacrine cells and PKCα, a marker of rod bipolar cells (G–H). The gross disorganization of cholinergic amacrine cell neurites observed in the *Dscam^2J^* retina is not observed in the *Dscam^3J^* retina, although limited examples of disorganization do occur (E; arrow). **I**–**K**, Wild type, *Dscam^2J^* and *Dscam^3J^* retinal sections were stained with antibodies to ChAT, a marker of starburst amacrine cells and Syt2, a marker of type 2 and type 6 cone bipolar cells (I–K). The wild type laminar specificity pattern of type 2 and type 6 cone bipolar cell axons is disrupted in the *Dscam^2J^* retina (arrowheads). The scale bar in (K) is equivalent to 112.5 µm.

Neurite lamination defects are observed in *Dscam^2J^* mutant mice. Antibodies recognizing amacrine, ganglion and bipolar cell types were used to assay the integrity of laminar specificity in the *Dscam^3J^* inner plexiform layer compared to that of wild type and *Dscam^2J^* retinas (Figure 3). *Dscam^3J^* neurites targeted to appropriate laminae, as observed in *Dscam^2J^ and Dscam^del17^* mutants (bNOS and cholinergica amacrine cells; A-C, dopaminergic amacrine cells and melanopsin positive RGCs; D-F and cholinergic amacrine cells and rod bipolar cells G-I). The lamination of *Dscam^3J^* neurites was more organized than that of *Dscam^2J^* retinal neurites, and significant deviations from wild type were not observed, although occasional examples of disorganized laminar specificity did occur (Figure 3G; arrow). Defects in laminar specificity of type 2 and type 6 cone bipolar cells, labeled with antibodies to synaptotagmin 2 (Syt2) were also observed in *Dscam^2J^* but not *Dscam^3J^* retinas (arrowheads). Diffuse Syt2 immunoreactivity was observed between cholinergic bands in the *Dscam^3J^* retina but this was distinct from the bipolar cell axons. Therefore laminar specificity is largely preserved in the *Dscam^3J^* retina.

**Figure 4:**
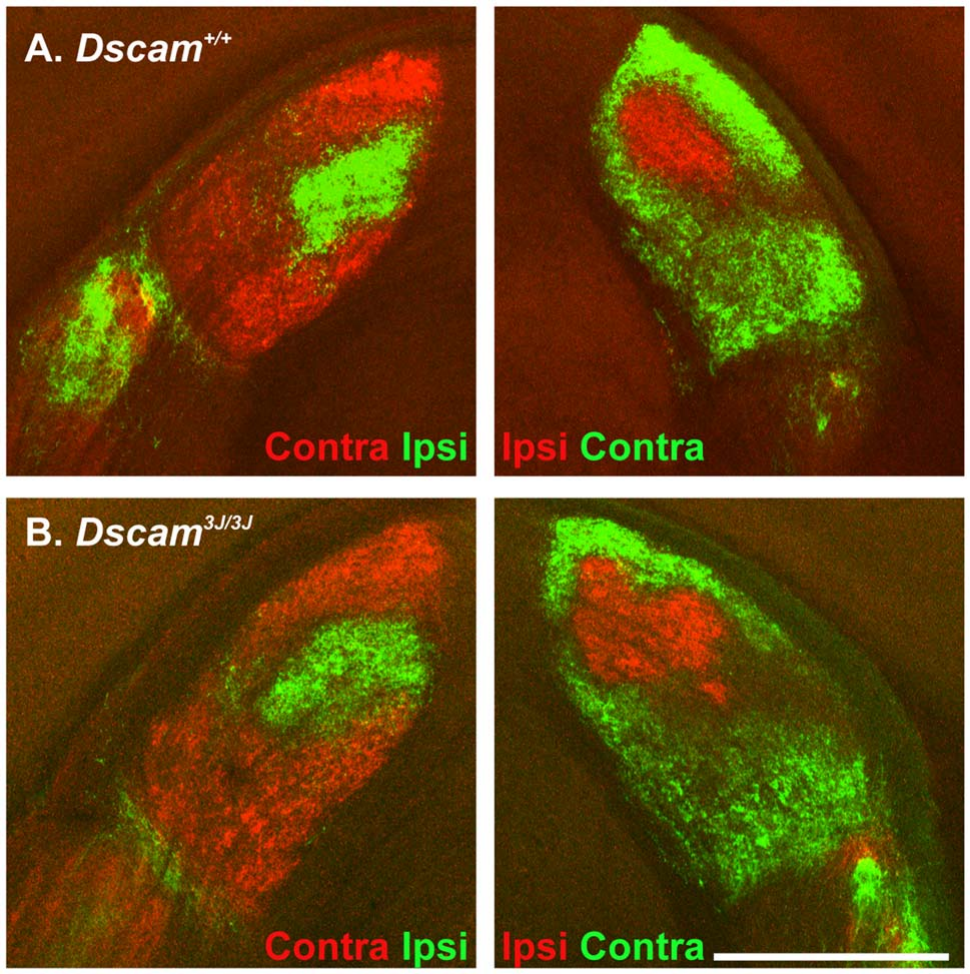
Normal LGN refinement in *Dscam^3J^* mutant. Cholera toxin labeled with alexa-488 or cy3 was injected into the right (cy3) or left (488) eyes of wild type and *Dscam^3J^* mutant mice to assay refinement of axonal projections. Both ipsilateral and contralateral projections of *Dscam^3J^* mutant mice were similar to wild type. The scale bar in (B) is equivalent to 225 µm.

Targeting and refinement of retinal ganglion cell axons has also been found to be *Dscam* dependent [18]. We therefore assayed the projection and eye specific segregation of ganglion cell axons to the dorsal lateral geniculate nucleus (LGN). Axons of retinal ganglion cells were labeled with cholera toxin conjugated to either alexa 488 or alexa 594, which was injected into the left or right eye, respectively. Projection of RGC axons to the LGN and segregation of ipsi and contralateral projections were comparable in the wild type and *Dscam^3J^* brain and did not resemble the disrupted patterning previously reported in *Dscam^del17^* mice, or other mutant alleles of *Dscam* (Figure 4; N = 4 and 6, respectively; data not shown) [18].

### Characterization of DSCAM and DSCAM^R1018P^

**Figure 5:**
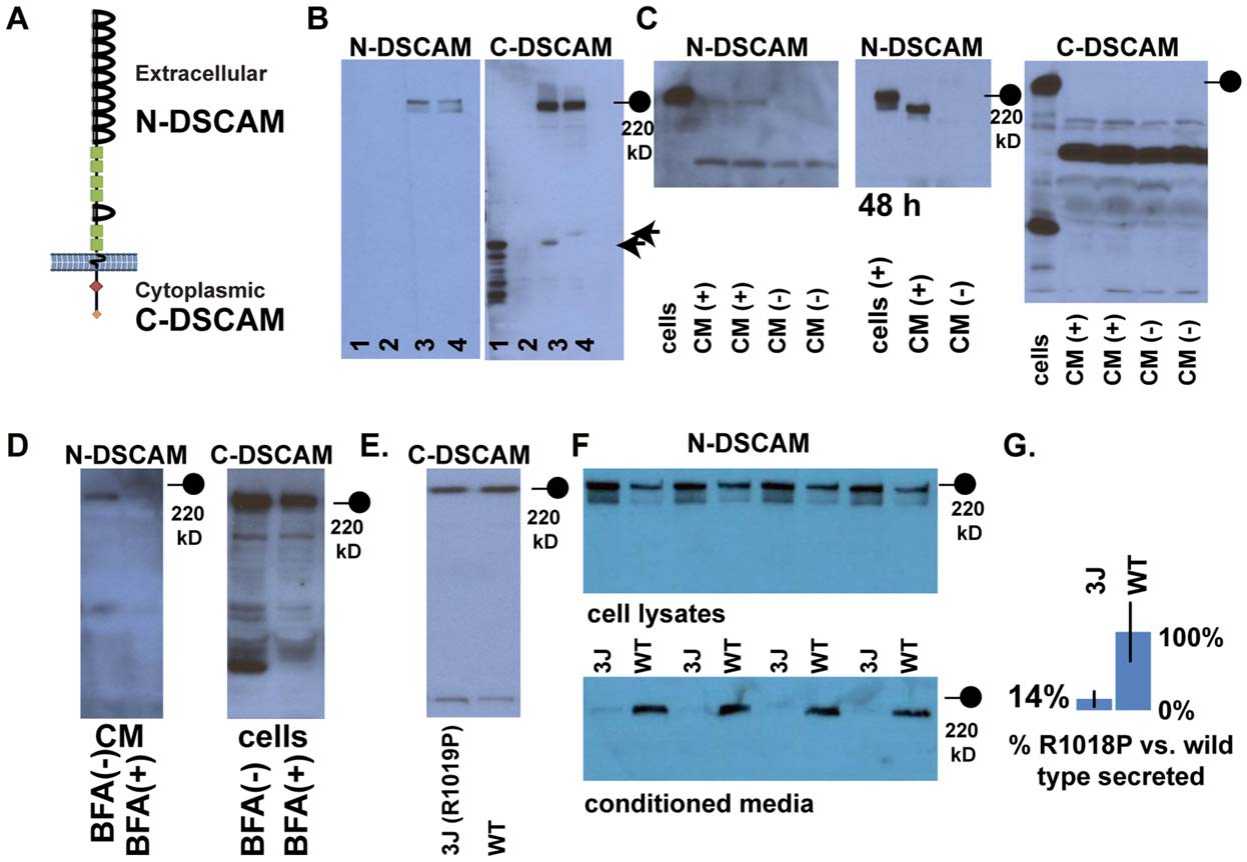
DSCAM is constitutively shed *in vitro* and the R1018P mutation inhibits this secretion. A , Antibodies recognizing the extracellular N-terminus of DSCAM (N-DSCAM) or the intracellular C-terminus (C-DSCAM) were used to monitor post-translational DSCAM processing. **B**, Lane 1; transfected with C-terminus of *Dscam*, lane 2; untransfected cells, lane 3; transfected with *Dscam* and lane 4; transfected with myc/his tagged *Dscam*. The N-terminal DSCAM antibody recognizes a band of approximately 220 kD and a band slightly smaller than 220 kD in lysates of DSCAM-transfected cells. The C-terminal DSCAM antibody recognized a band of approximately 220 kD and a band the same size of the DSCAM C-terminus, at about 45 kD (arrows). **C**, A band the same size as the smaller of the two bands recognized by the N-terminal DSCAM antibody was detected, and increases in abundance post-transfection, in conditioned media (CM) collected from cells transfected with *Dscam*, but not in CM from untransfected cells. This band is not detected by the C-terminal DSCAM antibody. **D**, Production of shed product is dependent on membrane trafficking, as addition of brefeldin A (BFA) inhibits production of shed product. Incubation of cells in brefeldin A (BFA) also inhibits production of the 45 kD band detected by the C-terminal antibody. **E** and **F**, Cell lysates and conditioned media from N2A or HeLa cells expressing either DSCAM or DSCAM^R1018P^ were analyzed. Wild type DSCAM protein was less abundant in cell lysates compared to DSCAM^R1018P^. A large decrease in the amount of shed DSCAM^R1018P^ was observed compared to shed wild type DSCAM. **G**, The amount of shed DSCAM or DSCAM^R1018P^ was normalized to the amount of protein observed in cell lysates. **H**, Models of DSCAM cleavage and secretion. DSCAM^R1018P^ is cleaved into two products but is not abundantly shed. This indicates that cleavage is not necessarily directly linked to secretion. This could be because cleavage occurs directly at the membrane and is linked to secretion and occurs separately after internalization. Alternately, cleavage could occur after internalization of DSCAM, the N-terminal portion of which is then secreted, and DSCAM^R1018P^ could be deficient in this subsequent secretion.

Based on histological analysis it appeared that the *Dscam^3J^* allele is capable of mediating a subset of DSCAM-dependent phenotypes. To better understand how the R1018P mutation interferes with DSCAM function, we characterized the expression and localization of wild type and R1018P DSCAM in vitro. *Dscam* expression constructs were generated that produce either the full-length canonical sequence of the *Dscam* open reading frame, or the cytoplasmic domain. Variants of these plasmids containing a C-terminal myc/his tag were also generated. Two antibodies raised against DSCAM were used to assay production of the protein in vitro; an antibody raised against the extracellular N-terminus of DSCAM and an antibody raised against the intracellular C-terminus (Figure 5A).

*Dscam* expression constructs were transfected into either HeLa cells or neuro-2A (N2A) cells Antibody specificity was tested by performing western blot analysis (WBA) on cells transfected with either the C-terminal *Dscam* construct, *Dscam* or myc/his tagged *Dscam* constructs compared to untransfected cells. The N-terminal DSCAM antibody recognized two bands of approximately 220 kD in cells transfected with *Dscam* (lane 3) or myc tagged *Dscam* (lane 4), but did not recognize bands in untransfected (lane 2) cells or cells transfected with the C-terminus of *Dscam* (lane 1) (Figure 5B). Blots were stripped and reprobed using C-terminal specific antibodies. The C-terminal DSCAM antibody recognized a smear of bands at and below 45 kD (the expected size of the DSCAM C-terminus) in lysates from cells that had been transfected with the C-terminus of *Dscam*. No band was detected in lysates from untransfected cells. Two bands from lysates transfected with *Dscam* or myc/his tagged *Dscam* were detected. The larger of these bands matched the size of full length DSCAM. The second matched the size of the C-terminus of DSCAM. The smaller band from myc/his tagged *Dscam* transfected lysates was shifted up in size slightly, confirming that this band is a product of the *Dscam* transcript (Figure 5B; arrows).

The presence of multiple bands produced from a single cDNA construct, and their respective sizes, suggested that DSCAM was being cleaved post-translationally into N-terminal and C-terminal fragments. Many cell adhesion molecules are processed by cleavage of their ectodomain and cytoplasmic domains after they reach the plasma membrane. Further studies were therefore conducted to investigate the mechanism by which DSCAM is processed. We first determined whether DSCAM is shed into cell culture media. WBA was performed on conditioned media (CM) from transfected and untransfected cells to determine if DSCAM was shed. N-terminal DSCAM antibodies labeled a band in conditioned media from transfected cells corresponding to the size of the N-terminus of DSCAM, while C-terminal antibodies did not recognize a specific band, indicating that the N-terminal portion of DSCAM can be shed. We also found that the amount of shed DSCAM protein increased over time (Figure 5C). To confirm that secretion was the result of an active process, occurring after DSCAM localization to the plasma membrane, and not the result of cell rupture, cells were treated with brefeldin-A (BFA), to block trafficking to the plasma membrane. Treatment with BFA effectively eliminated production of the shed isoform of DSCAM and production of the smaller C-terminal fragment of DSCAM, indicating that the protein must reach the plasma membrane before cleavage can occur (Figure 5D).

We next introduced the R1018P mutation into the *Dscam* expression construct and assayed expression by WBA. Expression of *Dscam^R1018P^* resulted in production of full length DSCAM and the small C-terminal band (Figure 5E). WBA of DSCAM^R1018P^ cell lysates indicated that both bands detected by the N-terminal antibody were present; however, very little DSCAM^R1018P^ was detected in conditioned media produced by the same cells (Figure 5F). The extent of DSCAM secretion was quantified by comparing the ratio of full length DSCAM detected in cell lysates using the N-terminal DSCAM antibody to the amount of DSCAM in conditioned media produced by the same cells. A significant decrease was found in the amount of DSCAM shed into conditioned media when comparing R1018P DSCAM to wild type DSCAM (Figure 5G).

**Figure 6:**
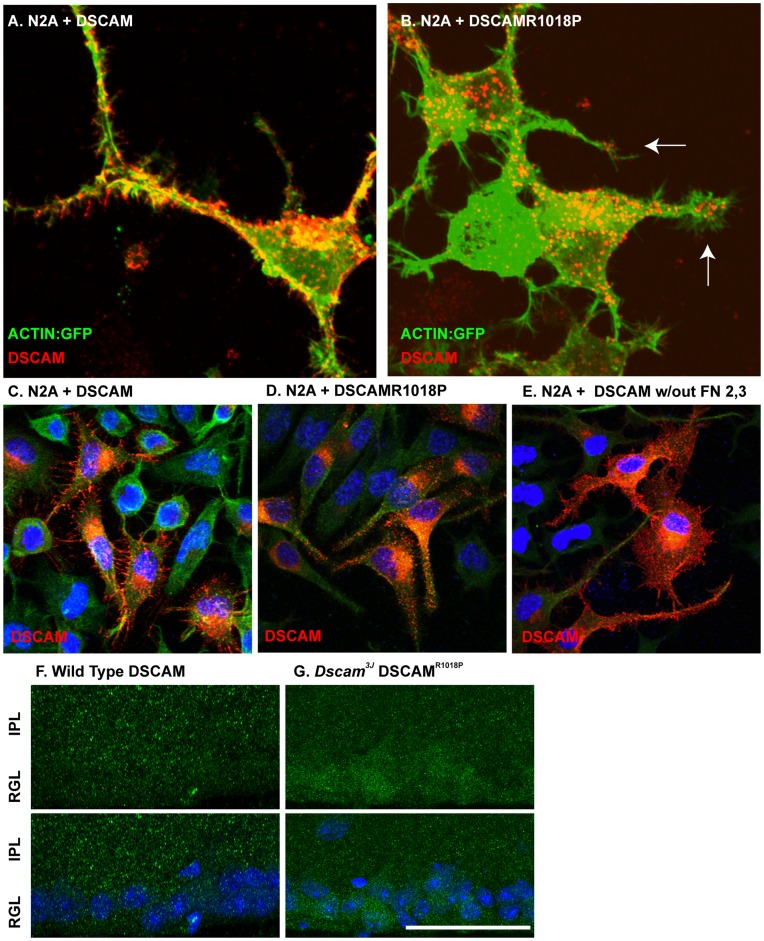
*Dscam^3J^* mutation inhibits filopodial localization of DSCAM. **A**–**C**, *Dscam* expression constructs were transfected into N2A Cells. **A**, Full length canonical DSCAM is abundant in the golgi-ER, where it is translated and trafficked, along the membrane and at the tips of filopodia. **B**, DSCAM^R1018P^ is translated and trafficked in and from the golgi-ER and appears in trafficking vesicles within growth cones but does not appear to be localized to the filopodia. **C**, The localization defect in DSCAM^R1018P^ is not a result of disrupted fibronectin domain function, as deletion of the second and third fibronectin domain of DSCAM does not interfere with filopodial localization. **D** and **E**, DSCAM or DSCAM^R1018P^ were transfected into N2A cells together with a plasmid expressing an actin-GFP fusion. DSCAM was easily visualized at the tips of actin filaments, whereas little DSCAM^R1018P^ was observed at the end of such filaments. Sections of wild type and *Dscam^3J^* retina were stained with antibodies to DSCAM. Cells of the *Dscam^3J^* retinal ganglion cell layer have an increase in immunoreactivity in their cell bodies compared to wild type. The scale bar in G is equivalent to 12.16 µm in A and B, 36.5 µm in C-E and 60 µm in F and G.

**Figure 7:**
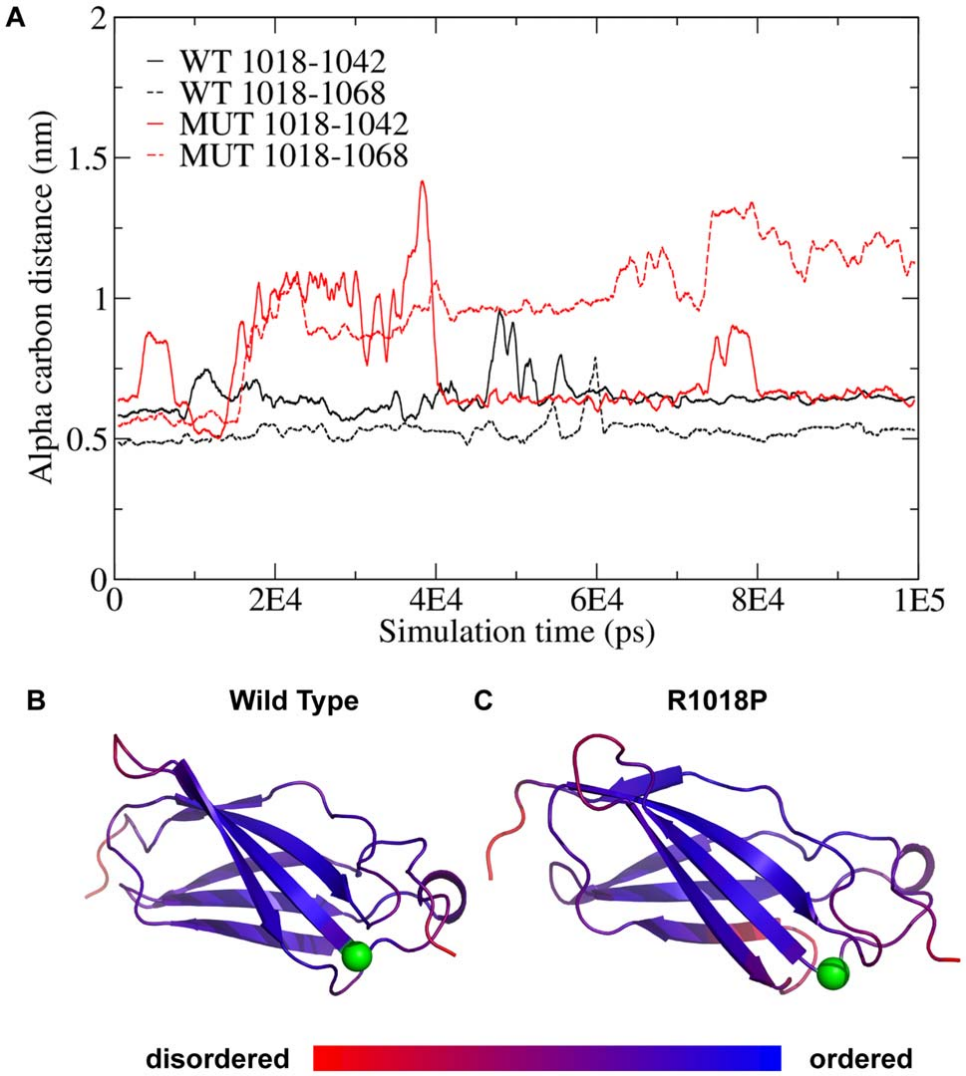
Molecular dynamics simulations suggest that the R1018P mutation destabilizes the DSCAM fibronectin domain. (A) Separation between the alpha carbon at the mutation site residue and two alpha carbons on neighboring loops as a function of simulation time. (B) Predicted structure of the wild type domain. The alpha carbon of amino acid 1018 is shown as a green sphere and the other colors represent the amount of fluctuation in the structure (blue = smallest fluctuations, red = largest fluctuations). (C) Same as panel B, but for the R1018P mutant.

Cells expressing *Dscam* or *Dscam^R1018P^* were stained with N-terminal antibodies to assay if a difference in localization could account for the *Dscam^3J^* phenotype and lack of DSCAM^R1018P^ secretion. DSCAM was localized to the fillapodia of N2A growth cones, while DSCAM^R1018P^ was localized to vesicles within the growth cone, with only small amounts of the protein visible in the filopodia (Figure 6 A and B). DSCAM^R1018P^ is located in the second fibronectin repeat of DSCAM. We generated a *Dscam* expression construct that lacks the second and third fibronectin repeats to assay if this domain is required for localization of DSCAM to filopodia. DSCAM lacking the middle two fibronectin repeats localized to filopodia, suggesting that the R1018P mutation does not prevent localization to the filopodia by disrupting the normal function of these repeats (Figure 6 C–E). The R1018P mutation occurs immediately before a predicted beta-strand, based on the structure of a type III fibronectin repeat from DSCAML1, which shows a high degree of homology [20]. Based on the abnormal localization in vitro we speculated the mutation might get trapped in the cell body in vivo. We therefore compared the localization of DSCAM and DSCAM^R1018P^ in sections of retina (Figure 6 F and G). While the DSCAM antibody used shows some nonspecific background, a clear increase in DSCAM^R1018P^ immunoreactivity is observed in the cell body of neurons in the retinal ganglion cell layer in *Dscam^3J^* sections compared to wild type DSCAM protein in wild type sections. Therefore the DSCAM^R1018P^ allele is not localized normally, but wild type localization in vitro is independent of the fibronectin repeat where the mutation occurs. This likely reflects larger scale changes to protein structure, which may be unsurprising given the unique conformation of proline and its effect on protein structure. To test this speculation we used molecular dynamics simulation to model the effect of the R1018P mutation on the structure and dynamics of the fibronectin domain. Figure 7A shows the separation between the alpha carbon at the mutation site and two alpha carbons on neighboring loops as a function of simulation time. Results show that the mutation increases the distance between these carbons, consistent with the idea that the mutation is destabilizing the protein domain. Figure 7 B and C shows the predicted structure of the wild type and R1019P DSCAM fibronectin domain based on homology modeling using a Dscam-like structure as a template. The alpha carbon of amino acid 1018 is shown as a green sphere and the other colors represent the amount of fluctuation in the structure (blue = smallest fluctuations, red = largest fluctuations). Results show that the mutation increases the dynamic fluctuations around the mutation site and is consistent with the idea that the mutation is destabilizing the protein domain.

## Discussion

In this study we describe a novel mutant allele of the mouse *Dscam* gene, *Dscam^3J^*. *Dscam* is one of many cell adhesion molecules that contributes to retinal development. *Dscam* stands out with respect to the larger number of phenotypes observed in *Dscam* mutant mice, including arborization, cell number and neurite laminar specificity defects. Understanding the relationship of defects observed in *Dscam* mutant mice, together with normal function of the DSCAM protein, remains a challenge. The results of this study will facilitate a better understanding of these processes.

Dscams have been most thoroughly studied in Drosophila, and mediate a similar range of processes as their vertebrate homologues, which lack the extensive alternative splicing observed in Drosophila Dscam1 [21], [22], [23], [24], [25], [26], [27]. Studies of vertebrate Dscams have identified at least three potential functions for these genes that overlap with functions demonstrated for their homologues in Drosophila. The first of these phenotypes, described in zebrafish, is a role in axon outgrowth [28]. Mouse DSCAM was subsequently found to bind the ligands netrin and draxin, although identifying guidance phenotypes has been uncertain [29], [30], [31], [32]. Next, gain of function and loss of function in chick suggest that Dscams are required for neurite laminar specificity [33]. Studies using mouse mutants have identified function in arborization, regulation of cell number and facilitating mosaic distribution of cell soma [12], [13].

Initial studies of mouse *Dscam* mutants found that wild type *Dscam* is required for normal spacing and arborization of amacrine cells. The different laminar targeting of these cell types suggested that DSCAM might act directly like MEGF proteins to facilitate avoidance. The finding that most retinal ganglion cells also require DSCAM to avoid getting entangled in like dendrites indicated that this model was too simple, because of the significant overlap of retinal ganglion cell dendrites. This suggested that mouse Dscams are acting as part of a larger identity code, and do not specify cell type [13]. Conditional deletion of *Dscam* confirmed that the protein acts to prevent fasciculation by acting within and not between retinal cell types and through homotypic binding, in that the null phenotype is dominant within cell types, that is mutant cells will entangle homotypic wild type cells within the same retina [34]. All of these mechanisms in mouse retina are consistent with DSCAM acting as a homophilic transmembrane cell adhesion molecule.

In this study we describe a new mutant allele of *Dscam*, further characterize the protein and show that DSCAM can be either shed or secreted. The *Dscam^3J^* allele shows a mixture of wild type and mutant phenotypes (Table 1). Unlike *Dscam^2J^*, in which no protein product was observed in retina or brain, *Dscam^3J^* makes a full-length protein. A simple explanation for the intermediate phenotypes observed in the *Dscam^3J^* allele is that the *Dscam^3J^* mutation is functionally a hypomorph, as a result of defective protein trafficking or reduced protein activity. Limited dopaminergic amacrine cell and ipRGC spacing disruption and cell number increases are previously described *Dscam* dosage phenotypes and the presence of these phenotypes in the *Dscam^3J^* retina is consistent with the allele acting as a hypomorph.

**Table 1:** Phenotypes in *Dscam^3J^* mice.

Phenotype	Wild type	*Dscam^2J^*	*Dscam^3J^*
Dendrites	Arborized	Fasciculated	Mixed
Soma Spacing	Spaced	Clumped	Weak Clumping
Cell number	Wild type	Overabundant	Overabundant
Neurite lamination	Organized	Disorganized	Organized
LGN refinement	Wild type	Abnormal	Like Wild type
In vitro secretion	Yes	N.A.	No
In vitro targeting	Filopodia	N.A.	No

Both wild type and DSCAM^R1018P^ get cleaved into two products in vitro; however, DSCAM^R1018P^ is shed in conditioned media at very low levels compared to wild type DSCAM. The lack of DSCAM^R1018P^ secretion seems best explained as the result of localization defects. Evidence for secretion of wild type DSCAM was found in vitro, where it is clearly secreted, and also in vivo, where the detection of a smaller isoform of DSCAM would be consistent with shedding. Secretion of fly DSCAM has also been reported but potential functions in neural development have not been identified [35]. In the context of DSCAM’s role in preventing adhesion, cleavage and secretion may be unsurprising as the tight binding of DSCAM isoforms must be overcome in order to facilitate repulsion, in flies, or prevent adhesion, in mouse, yet it has not been well documented [36]. Secretion and anchoring of DSCAM to cells in a similar fashion to the manner in which the axon guidance molecule Slit has recently been reported to act could explain why some cell types that do not express *Dscam* have a strong laminar specificity phenotype in *Dscam* mutant mice [37]. New studies taking advantage of newly developed conditional gain of function *Dscam* mouse alleles to complement existing mutant alleles will permit identification of potential roles of shed DSCAM in vivo by combining the ability to assay loss of function with conditional over or ectopic expression.

The contribution of cell adhesion molecules and the recognition they encode remains an emerging story in developmental neurobiology. In this study we identify a localization and secretion-deficient point mutant allele of mouse *Dscam* and use this model to better characterize the role of DSCAM in neural development. This resource will aid in efforts to understand how neural patterning is facilitated by the balance of adhesion and avoidance.

## Materials and Methods

### Animal Care and Ethics

All protocols were performed in accordance with the University of Idaho Institutional Animal Care and Use Committee which draws from NIH guidelines to ensure that animal suffering is minimized. Mice were fed ad libum under a 12-hour light/dark cycle. Mice taken for study were first deeply anesthetized with tribromoethanol (500 mg/kg). Blood was flushed out of vessels by cardiac perfusion. Following cardiac perfusion mice were decapitated and tissue was collected.

### Protein Extraction and Western Blot

Protein was extracted from tissue and separated into hydrophilic and hydrophobic fractions using mem-PER Eukaryotic Membrane Protein Extraction Reagent Kit according to instructions (Thermoscientific, Rockford, IL). Both fractions were found to contain DSCAM or DSCAM^R1018P^ and the cytoplasmic fraction was used because it contained more protein. All reagents included the addition of 0.005M EDTA and Halt protease and phosphatase inhibitor cocktail (Thermoscientific, Rockford, IL). Western blotting was performed as described previously [38] with the following modifications. Tissue protein samples were diluted two-fold to prevent band and lane distortion. Samples were incubated in 25% loading buffer for 1 hour at room temperature. Samples were not boiled in order to avoid agglutination of larger membrane proteins. Electrophoresis was performed on hydrophilic and hydrophobic fractions using 7% polyacrylamide gels. Transfer of proteins to immobilon-P membranes (0.45 um; Millipore, Billerica, MA) was carried out for 19–22 hours using 30 volts at 4°C. Methanol- free transfer buffer containing 0.037% SDS was used for the wet transfer to prevent precipitation of larger membrane proteins out of the gel. Membranes were blotted with a goat primary antibody directed against the N-terminus of DSCAM at 1∶500 and a Bovine anti-goat IgG-HRP secondary antibody (Santa Cruz Biotechnology, Santa Cruz, CA) at 1∶25000.

### Genotyping

Tissue was prepared for genotyping by boiling toe tip or tail tip biopsies in 25 µM sodium Hydroxide and 0.2 µM EDTA for 15 minutes. Samples were neutralized with an equal volume of Tris Cl, pH 5.0. DNA was added to OneTaq Hot Start 2X Master Mix with standard buffer (New England Biolabs, Ipswich, MA) and run with the following PCR cycle: 94°C 2 minutes, 38 cycles of 94°C 30 seconds, 53.5°C 30 seconds, 72°C 25 seconds, followed by a final 72°C incubation for two minutes. Primers used were 5′ CTT TGC GCG TTA TGA TC T 3′ and 5′ GTG GTG TCG ATA CTG ATG 3′. PCR products were incubated at 60°C for 2 hours in an equal volume of digestion mix containing 1 µl BstUI restriction enzyme, 10% Buffer 4 and then run on a 2% agarose gel for approximately 1.5 hours.

### cDNA Synthesis and RT-PCR

Total brain was homogenized in trizol reagent and RNA was isolated according to manufacturer’s instructions. A total of 1 µg total RNA was used to synthesize cDNA using a superscritpt III kit (Invitrogen) according to manufacturer’s instructions.

### Brain Section Staining

Brains were fixed in 4% paraformaldehyde overnight and sectioned at 150 µm with a vibratome. Brain sections were stained with 0.1% cresyl violet and 0.3% acetic acid for five minutes and then rinsed in PBS.

### Retina Staining

Eyes were enucleated following cardiac perfusion and hemisected. The posterior half of the eye was incubated in 4% paraformaldehyde for 50 minutes (DSCAM staining), overnight (for melanopsin staining) or 4–6 hours (all other staining) followed by three washes in PBS. For sectioning, retinas were isolated from the posterior half of the eye and either dehydrated and embedded in paraffin or equilibrated in 30% sucrose for one hour and then frozen in OCT. Sections were cut with a cryostat or microtome at 10 µm onto superfrost plus slides. Paraffin sections were rehydrated and stained with hematoxylin and eosin. Frozen sections were blocked in 7.5% normal donkey serum and 0.1% triton X-100 in PBS (block). Primary antibodies were diluted in block and incubated overnight at 4°C. Primary antibodies were washed three times in PBS for ten minutes. Secondary antibodies were diluted in block and 500 µl was applied over a given slide and incubated at room temperature for two hours. Slides were then washed three times for fifteen minutes in PBS. The second wash contained 1 µl DAPI solution. Whole retinas were stained in a similar fashion except that the block contained 0.4% triton and incubation of primary antibodies was carried out over four days, while secondary antibodies were incubated at 4°C for four days.

### Antibodies

Goat-anti-DSCAM (R&D Technologies 1∶500 for WBA and 1∶100 for IHC), rabbit-anti-melanopsin (generously gift of Ignacio Provencio, Uniformed Services University of the Health Sciences, at 1∶10,000), mouse-anti-tyrosine hydroxylase (Leica 1∶50), rabbit-anti-bNOS (Sigma Aldrich 1∶5,000), goat-anti-ChATp (Chemicon; 1∶500), mouse-anti-PKCα (Santa Cruz Biotechnology 1∶500), rabbit-anti-DSCAM (generous gift of Robert Burgess, The Jackson Laboratory) and mouse-anti-synaptotagmin2 (ZIRC; 1∶500). Fluorescent secondary antibodies were obtained from Jackson Immuno Research and were used at 1∶500.

### LGN Labeling

Mice were anesthetized with isofluorane gas. Following anesthesia, pupils were dilated by instillation of 1 drop of tropicamide 5%. One drop of tetracaine 1% was then administered for local anesthesia. Levels of anesthesia were monitored by brushing the cornea of the mouse with a fine paintbrush. Paralube was applied to eyes, removed during injection and reapplied following completion of injection in order to prevent eyes from drying out. Intravitreal injections were made using a 30 gauge Hamilton syringe. The eyes were visualized using a stereo dissecting microscope. A puncture was made in the eye posterior to the anterior/posterior boundary using the 30 gauge needle, which was inserted into the posterior chamber of the eye at a 45 degree angle in order to avoid the lens. Intravitreal injections of cholera toxin-β subunit (CTβ), conjugated to Alexa Fluor 488 (green) or Alexa Fluor 594 (red) were performed. CTβ (Invitrogen) conjugated to Alexa Fluor 488 (green label) was injected into one eye, and CTβ conjugated to Alexa Fluor 594 (red label) was injected into the other eye (2–3 µl; 0.5% in sterile saline). Placement of the needle within the eye and release of dye was monitored by directly viewing the posterior chamber of the eye through the stereomicroscope. Post-injection mice were placed in an incubator set to 30°C, until recovery and housed for 24 hours. 24 hours after injection, mice were euthanized and brain tissue was harvested and fixed overnight in 4% paraformaldehyde (PFA), cryoprotected in 30% sucrose, then sectioned coronally at 150 µm on a vibratome, mounted onto slides, and coverslipped with Vectashield (Vector Laboratories). Slides were imaged with an Olympus spinning disk microscope.

### Expression Constructs

The open reading frame of *Dscam* was PCR amplified out of total mouse brain cDNA and cloned into the EcoRI site of pCAG to generate pCAG-*Dscam*. A Myc/His tag was added to the C-terminus to generate pCAG-*Dscam^Myc/HIS^*. Site directed mutagenesis was used to introduce the R1018P mutation into pCAG-*Dscam* to generate pCAG-*Dscam^R1018P^*. Site directed mutagenesis was used to delete the second and third fibronectin domains of *Dscam* from pCAG-*Dscam* to generate pCAG-*Dscam* ^Δ*fn2–3*^. All constructs were sequenced to ensure the accuracy of the sequence and any subsequent changes.

### Cell Culture

N2A and HeLa cells were acquired from American Type Culture Collection and maintained in DMEM with glutamax (Invitrogen) supplemented with 10% fetal bovine serum. Cells were split by releasing cells using TryplE (Invitrogen). Cells were transfected with Fugene6 reagent at a ratio of 3 µl Fugene to 1 µg DNA (Roche). Brefeldin A (Sigma Aldrich) was administered at a dose of 2 ug/ml one hour after transfection. Cells were maintained for twenty four or forty eight hours after transfection and harvested for imaging or western blot analysis. For imaging, cells were seeded onto poly-D-lysine treated coverslips and stained as retinal sections were, except that primary antibodies were applied for two hours at room temperature. Mem-per reagent (Thermo Scientific) was used to extract protein from cells.

### Molecular Dynamics Simulations

Initial coordinates for the wild type and mutant protein domains were obtained using SWISS-MODEL with Protein Data Bank structure 1VA9 as a template [39]. Simulations were performed using the GROMACS 4.5.5 and the GROMOS 53A6 force field in a dodecahedral box containing approximately 18,500 SPC water molecules [40]. The charge was neutralized using sodium and chloride ions with an ion concentration of 0.15 mol/L. All bond distances containing hydrogen atoms were constrained using the LINCS algorithm. The temperature was maintained at 300 K using Langevin dynamics, the pressure was maintained at 1.0 atm using the Parrinello- Rahman algorithm. Van der Waals interactions were truncated using a cutoff of 1.4 nm, and electrostatics were treated using reaction-field with a cutoff of 1.4 nm. Both protein systems were first minimized using steepest descent for 1000 steps, followed by 1.0 ns of simulation with heavy atom restrained, followed by 1.0 ns of unrestrained simulation. Production simulations were then performed for 100 ns for each protein, generating a trajectory of conformation snapshots for each protein system. Protein fluctuations were determined by calculating the root-mean square fluctuations of the alpha carbons. This calculation is performed by first performing a least squares superposition of the structures in each trajectory and then computing the root-mean square deviation of the alpha carbons from their average positions.

## Supporting Information

Figure S1 **Alternative splicing of mouse** ***Dscam*** **. A**, Western blot analysis was performed on p0 wild type and *Dscam^del17^* mutant cytoplasmic protein extracts. Two bands were observed in wild type extracts, corresponding to the size of full length DSCAM and a slightly smaller band. *Dscam^del17^* protein extracts had two faint immunopositive bands, both slightly smaller than the corresponding wild type bands. Similar results were obtained for membrane extracts except that the *Dscam^del17^* protein bands were barely visible (data not shown). **B**, An alternative splice site was identified within exon 15 of canonical *Dscam*. The transcript made by use of the alternate acceptor site is not in frame and no corresponding protein was detected. **C**, In the context of the *Dscam^del17^* mutation the alternative acceptor site regains the *Dscam* open reading frame before hitting a stop codon, resulting in the absence of 94 wild type amino acids and the substitution of 54 alternative amino acids.
(TIF)
