# Supplementary figures and images for: A Novel Mouse Dscam Mutation Inhibits Localization and Shedding of DSCAM
Source: PLoS One. 2012 Dec 26;7(12):e52652. doi: 10.1371/journal.pone.0052652 (PMC3530462; doi:10.1371/journal.pone.0052652)

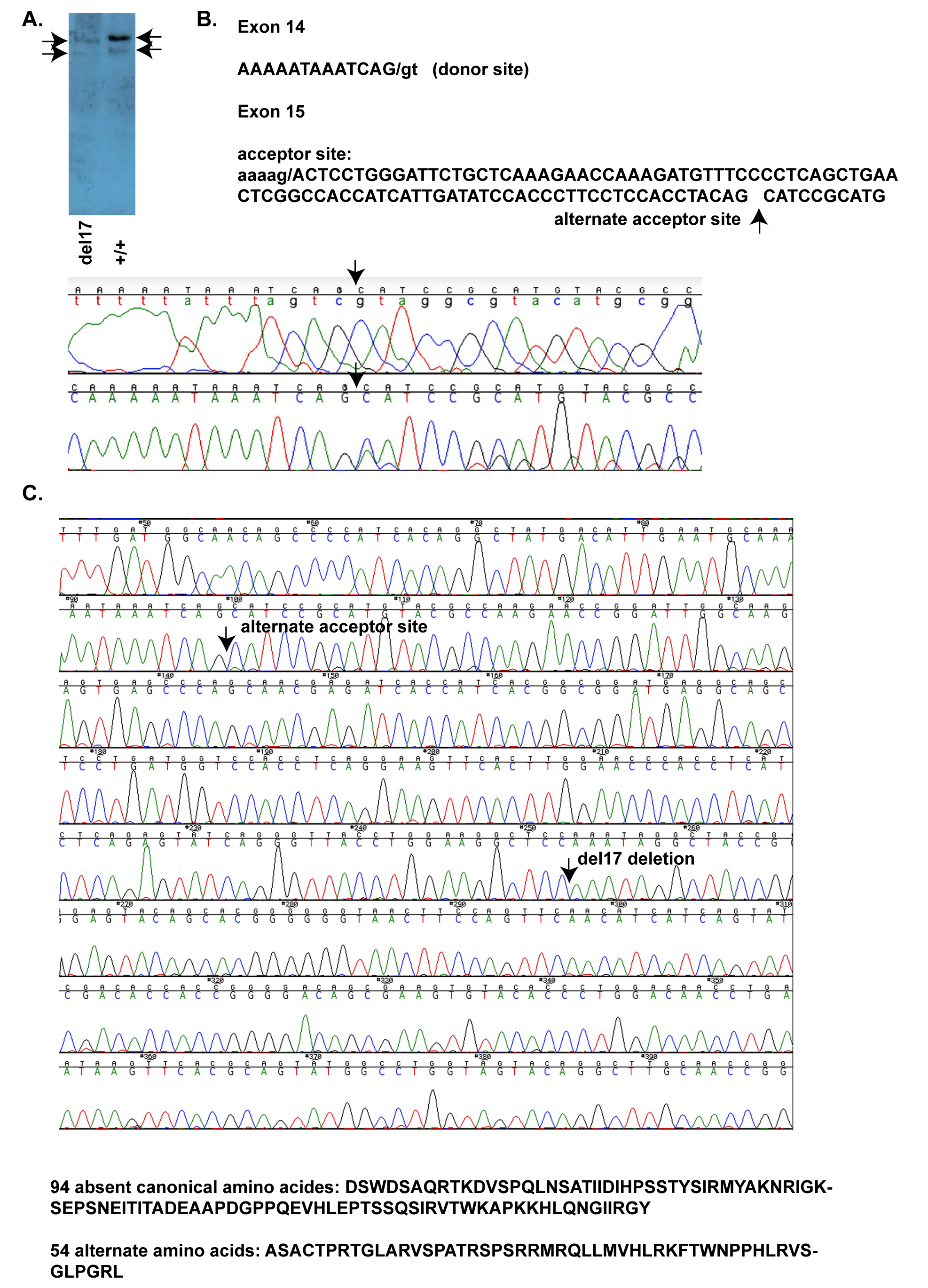

Supplement: Figure S1 — Alternative splicing of mouse Dscam . A, Western blot analysis was performed on p0 wild type and Dscamdel17 mutant cytoplasmic protein extracts. Two bands were observed in wild type extracts, corresponding to the size of full length DSCAM and a slightly smaller band. Dscamdel17 protein extracts had two faint immunopositive bands, both slightly smaller than the corresponding wild type bands. Similar results were obtained for membrane extracts except that the Dscamdel17 protein bands were barely visible (data not shown). B, An alternative splice site was identified within exon 15 of canonical Dscam. The transcript made by use of the alternate acceptor site is not in frame and no corresponding protein was detected. C, In the context of the Dscamdel17 mutation the alternative acceptor site regains the Dscam open reading frame before hitting a stop codon, resulting in the absence of 94 wild type amino acids and the substitution of 54 alternative amino acids. (TIF) [file pone.0052652.s001.tif]
